# Editorial: Engaging Undergraduates in Publishable Research: Best Practices

**DOI:** 10.3389/fpsyg.2019.01878

**Published:** 2019-08-27

**Authors:** Traci Giuliano, Jeanine Lee McHugh Skorinko, Marianne Fallon

**Affiliations:** ^1^Department of Psychology, Southwestern University, Georgetown, TX, United States; ^2^Department of Social Science & Policy Studies, Worcester Polytechnic Institute, Worcester, MA, United States; ^3^Department of Psychological Science, Central Connecticut State University, New Britain, CT, United States

**Keywords:** undergraduate students, research, psychology, publication, mentoring

## Introduction

As faculty members who are passionate about engaging undergraduates in publishable research, our goal for this Research Topic was to provide a forum for the dissemination of effective practices toward reaching this goal. We learned that we are not alone: Many faculty members throughout the world share our passion for collaborating with undergraduates on high-quality research. Although there is no shortage of books, articles, and resources^1^ on the topic of conducting research with undergraduates in psychology (see, for example, Developing, Promoting, and Sustaining the Undergraduate Research Experience in Psychology, Miller et al., 2008), far fewer articles have specifically addressed the process of publishing with undergraduates^2^. To address this gap, we assembled a large collection of articles that present effective models, innovative ideas, and solutions to the challenges of conducting publishable research with undergraduates. In doing so, we hope to increase the quantity and quality of such experiences worldwide. This Research Topic of Frontiers in Psychology contains 43 articles featuring 98 authors from the United States, Canada, Australia, and the United Kingdom who successfully engage undergraduates in publishable research. The diverse range of articles represented here (and summarized below) can be broadly grouped into five categories^3^: (1) structuring the curriculum to promote undergraduate research and publication, (2) optimizing research experiences for undergraduates, (3) training students in implementing advanced techniques, accessing special populations, or conducting research in off-campus settings, (4) addressing institutional and career challenges for faculty, and (5) increasing inclusion and diversity.

## Structuring the Curriculum to Promote Undergraduate Research and Publication

As nearly a dozen of the articles in the Research Topic illustrate, two of the best ways to facilitate publication with undergraduates are: (1) to structure the curriculum with multiple and often scaffolded opportunities for both skills training (e.g., in methodology, statistics, and writing) and faculty-student collaboration, and (2) to incorporate high-quality research projects with publication potential into specific courses.

### Curriculum

- Reavis and Thomas detail how their small liberal arts college's scaffolded psychology curriculum (which includes hands-on research in introductory courses, a combined statistics/methods course, a research-focused upper-level course, and a senior research course in which 2 faculty supervise 6–10 individual projects) provides multiple opportunities for faculty-student collaboration at every level and leads to presentations and publications.
- Canadian researchers McKelvie and Standing describe their department's 8-course methodology curriculum (which includes three semesters of statistics, a regular and an advanced methods course, a psychometrics/testing course, and a two-semester honors course) and two helpful practices (i.e., class discussion of published articles and replication projects) that have led to numerous undergraduate publications.
- In the first half of Mendoza and Martone, Mendoza highlights how the stepwise progression of his department's curriculum-oriented research experiences (i.e., CORE) helps turn their undergraduates into both critical consumers and producers of published research. CORE emphasizes developing critical thinking and writing skills, conducting original research in courses, and applying a graduate-school mentorship model to undergraduate research labs.
- Mickley Steinmetz and Reid describe how their psychology department incorporates research into most courses early and often to prepare students to produce publishable research. Upper-level core courses require original experiments designed for publication and a required apprentice-based senior thesis provides a capstone opportunity.
- Wieth et al. discuss their department's creativity-centered cooperative problem-solving (CPS) approach to undergraduate research and publication. The CPS approach exposes students to individual and group brainstorming, provides diverse feedback from students and faculty, and culminates in a senior thesis for the majority of students.
- Golding et al. describe the benefits (e.g., work readiness, networking, teamwork, publication) and challenges (e.g., mismatched expectations, equity in access) associated with the summer Undergraduate Research Experience at their Australian university.
- Because the United Kingdom has far fewer opportunities for undergraduates to present their research at conferences compared to the United States, Kent et al. suggest strategies to strengthen the pathway from undergraduate presentation to publication in the UK, including creating pre- and post-event community-building activities and incorporating conference presentation into the dissertation supervision process.

### Course Projects

- LoSchiavo describes seven helpful guidelines for incorporating a professional-grade, full-class project into a research methods course, which maximizes the probability of producing publishable undergraduate research.
- In her research methods course, Giuliano provides students with a “Writing Spiral” containing 10 handouts on grammar, citation, scientific writing, and APA style that has facilitated undergraduate publications in both research methods and capstone courses.
- Skorinko argues that project-based learning in courses can successfully lead to multiple forms of publishable scholarship, including the scholarship of discovery (e.g., research that advances knowledge), the scholarship of teaching (e.g., pedagogical articles), and the scholarship of engagement (e.g., applied and/or community service projects).
- Lineweaver and Bergeson outline eight steps for establishing a general education research course that turns non-science majors into civically-engaged student scientists who conduct a project in the community. They also discuss potential pathways to publication from this approach.

## Optimizing Research Experiences for Undergraduates

Over a dozen articles in the Research Topic are devoted to tailoring research experiences to undergraduate publication. These include articles that (a) describe effective practices (e.g., tips for recruiting and training students, the use of contracts and agreements, authorship discussions, writing weekends, open science practices), (b) recommend a variety of mentoring strategies, and (c) focus on the perspective of the undergraduate researcher.

### Effective Practices

- Scisco et al. provide a multi-site perspective based on their experiences at small liberal arts colleges, mid-sized regional universities, and a large research university; they offer strategies for selecting (e.g., targeting underrepresented groups and students with a growth mindset), managing (e.g., setting clear expectations and teaching collaborative writing skills), and engaging (e.g., by providing positive, instructive feedback and plenty of encouragement and support) undergraduate co-authors.
- Adams outlines four learner-centered practices that she has found effective in guiding undergraduates to produce publishable research, including building rapport, providing structure and clear expectations, teaching writing skills, and engaging in self-reflection.
- Bloomfield et al. present the content of their faculty-student co-authored “research agreements,” which they argue promote the ideal atmosphere for producing publishable research because such agreements foster discussions about scientific professionalism, ownership, and ethics and also allow faculty and students to reflect on shared project goals.
- Giuliano describes her experiences guiding students through the process of first authorship, including perceived barriers, paths to undergraduate first authorship, and best practices, including how to assign authorship order and credit.
- Scherman highlights the factors that promote successful “student writing weekends,” which address many of the barriers to undergraduate publication (e.g., continuous access to mentor instruction and feedback, dedicated space and time to write, freedom from work and family distractions) while providing a fun, bonding, and collegial experience.
- Strand and Brown discuss the benefits of using open science practices in their research labs and outline the steps they use in conducting publishable open science research with undergraduates.
- Wagge et al. also emphasize the advantages of conducting open science research with undergraduates, as illustrated by the high quality, publishable replications (from methods and capstone courses, as well as research labs) that have resulted from the Collaborative Replications and Education Project.

### Mentoring Strategies

- Holmes and Roberts compare the relative merits of four popular faculty mentoring models (*sculptor, makeover artist, coach, CEO*), arguing that the first two are superior for producing publishable undergraduate research characterized by high student interest and equitable faculty-student benefits.
- Overman describes three strategies for group-level mentoring of undergraduates (creating a shared vision, using interlocking projects, and building a lab community with strong relationships) that increase faculty productivity and lead to publications and grant funding.
- Detweiler-Bedell and Detweiler-Bedell advocate for a team-based approach that enhances students' sense of belonging and leads to student co-authored publications and external funding. In their model, laddered teams consist of an experienced senior lab member, a mid-level sophomore or junior lab member, and a student “assistant” new to the lab.
- Dunbar uses a similar team-based, peer mentoring approach at an R2 university, where graduate students participate in the mentoring teams and conduct parallel projects that, when combined with undergraduate projects, yield stronger multi-experiment papers and increase the number of undergraduate co-authors.

### The Undergraduate Perspective

- Matthews and Rosa, who recently graduated from a liberal arts university with Bachelor's degrees, reflect back on their research lab experience. They discuss the benefits (e.g., confidence, work ethic, critical thinking, graduate school/career preparation, presentations, and publications) and challenges (e.g., interpersonal dynamics, procrastination, project work continuing after graduation) of their research experience, along with tips for success (e.g., individual brainstorming prior to group brainstorming; meeting notes, task lists, and weekly progress reports; peer review and section-by-section writing/revising of drafts).
- In the second half of Mendoza and Martone, recent graduate Martone describes the impact that joining a faculty-led research lab had on her self-confidence, research and writing skills, and graduate school preparation, attributing her successful outcomes to the support and role modeling of her faculty mentor.
- Skorinko, a faculty member, outlines nine helpful strategies that are informed by her own experiences as an undergraduate for engaging students at her R2 institution in publishable research.
- In a qualitative interview study examining Australian undergraduates' perceptions of their dissertation experience, Roberts and Seaman found that helpful supervisors were supportive, directive, and had styles and interests that matched their students, whereas less helpful supervisors failed to provide clear expectations and/or treated students inequitably.

## Training Students in Advanced Techniques, Special Populations, or Off-campus Settings

Nearly a dozen articles in the Research Topic discuss the challenges of teaching undergraduates technically-advanced research skills (e.g., physiological assays, narrative research, archival research), working with special populations (e.g., children, primates, dolphins, rats), and conducting research in different settings (e.g., off-campus local research sites, community service, international field studies).

- Bukach et al. describe their PURSUE (Preparing Undergraduates for Research in STEM-related fields Using Electrophysiology) initiative, which incorporates cross-institutional collaboration and shared resources to address the challenges of publishing with undergraduates in cognitive neuroscience (e.g., working with complex technologies, such as EEG and ERP, that require time-intensive training).
- Goldey et al. similarly tout the benefits of cross-institutional collaboration for sharing resources, time, and expertise in conducting research on salivary biomarkers (e.g., cortisol, opioids) linked to physical and mental health. They offer step-by-step recommendations for others wanting to conduct such research.
- Because conducting clinical psychiatric research with undergraduates can be challenging (especially at universities without access to clients), Hammersley et al. recommend the use of both cross-disciplinary and cross-institutional collaboration to pool funds, equipment, supplies, and research assistants, as well as the use of publicly-available archival datasets to study clinical topics.
- Dunbar describes how the development of the Faculty for Undergraduate Neuroscience (FUN) organization, along with NSF funding and a partnership with a pharmaceutical company, increased his department's ability to conduct publishable neuroscience research with undergraduates.
- Grysman and Lodi-Smith discuss the challenges and best practices for conducting publishable narrative research (a mixed-method approach that involves qualitative coding of typed or spoken words prior to quantitative analysis) with undergraduates.
- Childers and Phillips describe strategies for conducting publishable research with undergraduates using advanced technical skills at off-campus research sites. Childers, a developmental psychologist, uses eye-tracking technology to study pre-school-aged children at local childcare centers; Phillips, a behavioral neuroscientist, uses neuroimaging to study non-human primates at the National Primate Research Center.
- In Mickley Steinmetz and Reid, Reid describes how her students produce publishable research in a single semester (with projects that involve training on a computer program and using rats as subjects) as part of her Learning and Adaptive Behavior course.
- Ashdown suggests five strategies for conducting publishable research with undergraduates abroad based on his past experiences in Guatemala: establish local connections, avoid superficial cultural understanding, secure institutional support for students, understand students' research and cultural skills, and model good international research ethics.
- Hill and Karlin share their best practices for stimulating publication-quality projects by underrepresented undergraduates enrolled in their research immersion program. This program consists of a two-semester course supplemented by a week-long international field experience involving research in comparative psychology or conservation biology using dolphins or other marine species.
- Burns-Cusato and Cusato, who conduct field research on green monkeys in Barbados, describe the benefits and challenges of collaborating with undergraduates abroad, and outline two models that can lead to high quality, publishable projects: faculty securing funding for a stand-alone research project or faculty teaching a study abroad research-based course.
- Mello-Goldner describes the lessons learned from her very small college's decade-long experience collaborating with community partners on research that benefits both the local community (in terms of understanding local issues) and her students (who gain access to larger datasets than they would have otherwise, obtain real world/applied experience, network for future internship and job opportunities, and cultivate a sense of civic engagement).

## Addressing Institutional and Career Challenges

Although a majority of the articles in the Research Topic are written by faculty at primarily undergraduate institutions that provide at least some support and reinforcement for publishing with students, a number of articles provide helpful suggestions for publishing with undergraduates at different types of institutions (including large research universities, regional universities, and community colleges) and for coping with other challenges related to resources, position, or career stage that could impact faculty wherever they teach.

- Lundwall et al. offer guidelines for balancing undergraduate- and graduate-student needs in publishing at an R1 university, including tips for recruitment, preparation, writing, and a modified peer-mentoring structure.
- Dunbar's article relates two strategies to maximize the involvement of undergraduates in publishable research at an R2 university, including the development of a cross-institutional faculty networking group in neuroscience and the use of both graduate and undergraduate student peer mentoring.
- Skorinko, whose institution recently moved from an R3 to an R2 classification, outlines nine helpful strategies for engaging undergraduates in publishable research in this environment.
- Dutta et al. describe three phases (cultivate and motivate, identify and select, polish and enhance) in the research journey with undergraduates at a regional university that can transform them from consumers to producers of publishable research.
- Frohardt, an administrator and neuroscience researcher at a community college, examines several ways to successfully engage community college students in publishable research, including seeking funding opportunities, prioritizing experiential learning, scaffolding students toward publishable research, and encouraging and recognizing strong mentorship.
- Wood, a faculty member in a teaching-focused position at a large Canadian research university, describes how “teaching stream” faculty (whose labs may consist of only undergraduate students) can produce rigorous, high quality research by relying on undergraduate research leadership.
- Mendoza and Golden, a pre-tenure faculty member at a primarily teaching institution and a tenured faculty member at a large research university, respectively, share their unique perspectives on how to overcome obstacles to publishing with undergraduates faced by faculty at different career stages and institution types.
- Stefanucci describes two strategies for preserving faculty time in a way that maximizes efficiency, ensures productivity, and is rewarding for both faculty and students: mentoring and delegating research tasks, and seeking credit (including course credit) from one's institution for mentoring and publishing with undergraduates.
- In their discussion of how their department's curriculum prepares undergraduates to publish, Mickley Steinmetz and Reid also note that a key feature of their success in publishing with students is that faculty-student research is incorporated into their standard faculty course load.

## Increasing Inclusion and Diversity

Perhaps no issue regarding student research is more important than increasing inclusion and diversity within our labs and providing equal access to publishing opportunities for all students. Psychology's “representation problem” (Peifer) occurs at every level: Underrepresented populations are less likely to participate in high-impact practices as college students (Stebleton and Soria, 2012), less likely to be undergraduate co-authors (Grineski et al., 2018), less likely to enter and complete doctoral programs in psychology (Callahan et al., 2018), and much less likely to publish in general (comprising 88% of the world's population but authoring only 20% of published articles; Henrich et al., 2010). A slew of articles in this Research Topic focus wholly or significantly on the goal of increasing diversity in undergraduate research.

- Peifer provides historical context for this issue, examining how three interconnected facets of diversity (racial, socioeconomic, and family educational history) influence undergraduates' engagement with the research and publication process. She also suggests general strategies that faculty mentors can employ to increase the diversity of perspectives not only in undergraduate publication, but in the field of psychology as a whole.
- Ahmad et al. outline several evidence-based strategies for promoting an inclusive research lab, including steps for recruiting (e.g., proactive strategies designed to attract students from all backgrounds), selecting (e.g., making efforts to recognize and minimize the impact of implicit biases), and retaining diverse students through strong mentorship.
- Chan recommends a “systems mapping” approach to create a positive and inclusive climate from the outset that culminates in publishable research for first-generation, historically underrepresented, and low-income undergraduates. This approach involves mapping the current lab or research team climate, monitoring participation and retention rates, and planning strategically throughout the research process.
- In their article describing the benefits of using laddered peer-mentoring undergraduate teams, Detweiler-Bedell and Detweiler-Bedell make the case that the enhanced sense of belonging created by such teams is especially beneficial for the recruitment, retention, and success of students from underrepresented groups.
- Dutta et al.'s article on transforming undergraduates at a regional university from consumers to producers of research considers the unique challenges faced by a student body that includes many first-generation college students, students who work long hours, and non-residential students.
- Frohardt's article examines ways that community college faculty, who often deal with heavy teaching loads and a reduced emphasis on scholarship for tenure and promotion, can increase opportunities to engage our most underrepresented students in publishable research.
- Hill and Karlin, faculty members at a Hispanic-Serving Institution in Texas, describe the challenges and best practices for conducting research (including an international field study experience) with underrepresented undergraduates.

## Conclusion

In closing, we are amazed by the collective–and in many cases collaborative–excitement, passion, and deep thought that so many colleagues around the world have put into conducting publishable research with undergraduate students. Clearly these efforts pay off, as evidenced by the authors who reflected on the importance of these undergraduate experiences in their educational and professional pathways. We believe that there is something for everyone interested in publishing with undergraduates in this Research Topic, as each article provides a unique and diverse perspective that nevertheless resonates across contexts and situations. We hope that the ideas, models, techniques, and practices in these 43 articles will motivate and inspire readers to begin, continue, or rethink how they engage undergraduates in high quality, publishable research. Finally, we hope to stimulate empirical and quantitative research (which is presently lacking) on the effectiveness of these ideas, models, techniques, and practices. We hope you learn as much from reading this issue as we have from curating it.

## Author Contributions

TG wrote the first draft. JS and MF provided comments for revision. All authors approved this article for publication.

### Conflict of Interest Statement

The authors declare that the research was conducted in the absence of any commercial or financial relationships that could be construed as a potential conflict of interest.

## Footnotes

^1^The Council on Undergraduate Research has an up-to-date, online compendium of excellent publications and resources on topics ranging from broadening participation in undergraduate research to models of undergraduate research in the arts and humanities to the impact of undergraduate research on student learning to excellence in mentoring student research (see https://myaccount.cur.org/bookstore/).

^2^For notable exceptions in psychology, see Wendt (2006), Cramblet Alvarez (2013), Hughes (2014), Hartley (2014), and Griffiths (2015); for other fields, see Burks and Chumchal (2009; biology), Bliss (2002; nursing), and Jalbert (2008 accounting).

^3^Some articles are mentioned more than once because they make significant contributions in more than one category.
